# Indoor Positioning and Navigation

**DOI:** 10.3390/s21144793

**Published:** 2021-07-14

**Authors:** Simon Tomažič

**Affiliations:** Faculty of Electrical Engineering, University of Ljubljana, 1000 Ljubljana, Slovenia; simon.tomazic@fe.uni-lj.si; Tel.: +386-1-4768-760

Recently, the social and commercial interest in location-based services (LBS) has been increasing significantly. The scientific field of indoor localization and navigation has undergone rapid development due to many studies considering advanced mobile and communication technologies [1]. The purpose of developing new localization algorithms and navigation systems is to enable autonomous mobile systems [2] to use these solutions in performing a specific task or to assist people who have lost their ability to navigate [3] (e.g., blind people, people with Alzheimer’s disease). A secure, user-friendly, and accurate indoor positioning system (IPS) that can run on a smartphone could open the door to many innovative applications and create new business opportunities. The global indoor location market is expected to reach a worth of $40.99 billion by 2022 [1]. A low-cost real-time locating systems (RTLS) are very useful as they can guide people through airports, shopping malls, museums, etc. [1], or improve production control and logistics [4]. Modern smartphones are equipped with numerous sensors (inertial sensors, camera, barometer) and communication modules (WiFi, Bluetooth, NFC, LTE/5G, ultra-wideband), which enable the implementation of different localization algorithms, namely visual localization, inertial navigation system and radio localization [1]. For the mapping of indoor environment and localization of autonomous mobile systems, LIDAR sensor is also commonly used besides smartphone sensors [2,5,6]. Since visual localization and the inertial navigation systems (INS) are sensitive to external disturbances, the sensor fusion approaches based on Kalman filters and (deep) neural networks can be used to implement robust localization algorithms, as proposed in [3,7]. The localization algorithms need to be optimized in order to be computationally efficient, which is essential for real-time processing and low energy consumption on a smartphone or robot. A practical indoor positioning system (IPS) should have characteristics such as ease of implementation, acceptable localization accuracy, scalable system, feasible system cost and minimal computational complexity.

In general, there are two main approaches to indoor localization, namely infrastructure-based and infrastructureless approaches [1]. The latter generally uses fingerprints of environmental features such as sound, light, magnetic field, or is based on smartphone sensors (e.g., accelerometer, gyroscope, etc.). Infrastructure-based methods can use pre-installed visual sensors or wireless technologies such as ZigBee, WiFi, Ultra-Wideband (UWB) [2,6], Radio Frequency Identification (RFID) [5], and Bluetooth Low Energy (BLE) [1]. An infrastructure-based indoor positioning system can be expensive, either because of the methods required or because of the expensive hardware components. Installing the necessary infrastructure is often time-consuming and labor-intensive, which drives up the price. To solve this problem, authors S. Tomažič and I. Škrjanc [8] developed an automated indoor localization system that combines all the necessary components to realize low-cost Bluetooth localization with the least data acquisition and network configuration overhead. The proposed system incorporates a sophisticated visual-inertial localization algorithm for a fully automated collection of Bluetooth signal strength data. The visual-inertial SLAM algorithm, which is part of the ARCore library, successfully fuses information from the camera and inertial sensors to provide accurate localization in large spaces over a long period of time. A suitable collection of measurements can be made quickly and easily, clearly defining which part of the space is not yet well covered by measurements. An automated system has many advantages in various approaches to data collection, e.g., quick and easy construction of datasets for model development or fingerprinting, beacon parameter studies, in-depth studies of beacon location and density in a given environment, and quick maintenance of the database as the environment changes [8].

Radio-based indoor positioning can generally be divided into three groups: Proximity, Time of Flight (TOF) measurements [9], and Received Signal Strength Indicator (RSSI)-based methods [10]. In some cases, these were also supplemented with an Angle of Arrival (AOA) localization approach [11]. Depending on the localization approach chosen to compute the current position, the following methods are most commonly used: triangulation, trilateration, and fingerprinting. Among these, fingerprinting is perhaps the most popular due to its simplicity: it is based on signal strength (RSSI) and its procedure is essentially to collect the signal from the transmitters and assign it to a specific position [1]. Since the RSSI suffers from low stability due to interference with objects and environmental effects, the authors Shi et al. [10] proposed a tri-partition RSSI classification and its tracing algorithm as an RSSI filter, which enables lower variance range. Using the filter improves the accuracy of trilateration-based positioning by 20.5%. In recent years, many commercial WiFi devices support the collection of physical layer channel state information (CSI). CSI is an index that can characterize signal characteristics with finer granularity than RSSI. Compared to RSSI, CSI can avoid the effects of multipath and noise by analyzing the properties of multichannel subcarriers. To improve the indoor location accuracy and the efficiency of the algorithm, Wang et al. [12] proposed a hybrid fingerprint location technology based on RSSI and CSI. The accurate hybrid fingerprint database was constructed after the dimensionality reduction of the obtained high-dimensional data values. Weighted k-nearest neighbor (WKNN) algorithm was applied to reduce the complexity of the algorithm during the online positioning phase.

BLE is one of the most widely used technologies in ubiquitous computing and many Internet-of-Things (IoT) applications because it offers many advantages such as low power consumption and low cost. As 5G technology is rising across the world and UWB chip is available in the latest smartphones, IPS can leverage and integrate these technologies in the future to develop a better IPS [1]. Authors Blaszkiewicz et al. [13] presented an interesting positioning solution, which utilizes existing radiating cables in tunnels or corridors without the need to deploy a dense network of reference nodes. Radiating cables are mostly used to provide radio communication in tunnels or corridors, but can also be used to estimate the position of a mobile terminal along the cable.

To improve indoor localization based on the time-of-arrival (TOA) principle, Deng et al. [9] proposed a clock synchronization solution for dynamic networks called Multi–Gaussian Variational Message Passing (M-VMP) method. The latter improved the positioning accuracy and convergence speed for mixed Line-of-Sight (LOS) and Non-Line-of-Sight (NLOS) environments. To optimize the anchor node density in TOA positioning approach the authors Deng et al. [14] developed a location source optimization algorithm based on fuzzy comprehensive evaluation. Authors Groth et al. [11] went one step further and developed a calibration-free single-anchor indoor localization method, including a dedicated algorithm and all necessary hardware modules. They showed that a single base station equipped with an ESPAR antenna to perform the measurements could be used to find the position of an unknown BLE tag without calibration or recalibration, since low-cost reference modules installed on walls inside the test area provided enough reference information for the positioning algorithm.

Global navigation satellite systems (GNSS) have long been employed for LBS to navigate and provide accurate and reliable location information in outdoor environments. However, GNSS signals are too weak to penetrate buildings and unable to provide reliable indoor LBS. In order to overcome this problem, authors Uzun et al. [15] proposed an indoor positioning system using Global Positioning System (GPS) signals in the 433 MHz Industrial Scientific Medical (ISM) band. The proposed method is based on down-converting (DC) repeaters and an up-converting (UC) receiver. The repeaters receive outdoor GPS signals at 1575.42 MHz (L1 band), down-convert them to the 433 MHz ISM band, then amplify and retransmit them to the indoor environment. When GPS signals at 433 MHz are received by the up-converting receiver, it amplifies these signals and up-converts them back to the L1 frequency. Then, the commercially available GPS receiver calculates the pseudo-ranges.

In the field of indoor localization, visible light positioning (VLP) systems are also very promising. Authors Amsters et al. [16] presented an innovative solution for calibrating VLP systems using a mobile robot to facilitate data acquisition. The new approach significantly improved performance compared to previous studies, almost doubling the accuracy of LED localization. The authors showed that the ambient illumination had little impact on the proposed method. Authors Jaenal et al. [17] developed an appearance-based robot localization in 2D with a sparse, lightweight map of the environment consisting of descriptor–pose image pairs. The authors proposed a piecewise approximation of the geometry of descriptor manifold through a tessellation of the so-called Patches of Smooth Appearance Change (PSACs), which defines their appearance map. The authors’ proposal is based on the assumption that the global image descriptors form a manifold articulated by the camera pose that adequately approximates the Image Manifold. On the map, the presented robot localization method applies both a Gaussian Process Particle Filter (GPPF) to perform camera tracking and a place recognition technique for re-localization within the most likely PSACs according to the observed descriptor.

Due to the benefits of indoor positioning technology, numerous indoor navigation applications have been deployed in large buildings, such as hospitals, airports, and train stations, to guide visitors to their destinations. A commonly used user interface displayed on smartphones is a 2D floor map with a route to the destination. Navigation instructions, such as turn left, turn right, and go straight on, are displayed on the screen when the user arrives at an intersection. However, due to the limitations of a 2D navigation map, users may be under mental pressure and become confused while making a connection between the real environment and the 2D navigation map before proceeding. For this reason, authors Huang et al. [18] developed ARBIN, an augmented reality-based navigation system that displays navigation instructions on the real environment screen for ease of use. The positions are determined using BLE beacons and RSSI models.

Several indoor localization solutions are already being used in practice or are ripe for implementation in real-world environments. Nevertheless, there is still much room for development of new approaches and standards in the field of indoor localization. Hopefully, these new standards will make new solutions as useful as GNSS is for outdoor localization and navigation. 
